# Glibenclamide Induces Collagen IV Catabolism in High Glucose-Stimulated Mesangial Cells

**DOI:** 10.1155/2012/183535

**Published:** 2012-09-12

**Authors:** Liping Zhu, Pedro Cortes, Clare Hassett, David W. Taube, Jerry Yee

**Affiliations:** Division of Nephrology and Hypertension, Department of Medicine, Henry Ford Hospital, Detroit, MI 48202, USA

## Abstract

We have shown the full prevention of mesangial expansion in insulin-deficient diabetic rats by treatment with clinically-relevant dosages of glibenclamide (Glib). Studies in mesangial cells (MCs) also demonstrated reduction in the high glucose (HG)-induced accumulation of collagens, proposing that this was due to increased catabolism. In the present study, we investigated the signaling pathways that may be implicated in Glib action. Rat primary MCs were exposed to HG for 8 weeks with or without Glib in therapeutic (0.01 **μ**M) or supratherapeutic (1.0 **μ**M) concentrations. We found that HG increased collagen IV protein accumulation and PAI-1 mRNA and protein expression, in association with decreased cAMP generating capacity and decreased PKA activity. Low Glib increased collagen IV mRNA but fully prevented collagen IV protein accumulation and PAI-1 overexpression while enhancing cAMP formation and PKA activity. MMP2 mRNA, protein expression and gelatinolytic activity were also enhanced. High Glib was, overall, ineffective. In conclusion, low dosage/concentration Glib prevents HG-induced collagen accumulation in MC by enhancing collagen catabolism in a cAMP-PKA-mediated PAI-1 inhibition.

## 1. Introduction

We have reported the complete prevention of glomerular mesangial expansion and increased albuminuria by the long-term administration of sulfonylureas in streptozotocin-diabetic rats [1]. This prevention was induced by low drug dosages glibenclamide (Glib, or glyburide) 0.28 mg/Kg (0.04 mg/Kg if adjusted for differences in drug clearance rates between rats and humans [2]) as compared to those clinically administered (0.018–0.286 mg/Kg). The protective effect occurred without improvement in glycemia, renal hypertrophy, or glomerular hyperfiltration. Thus, the reasons for these unexpected findings were not apparent.

One of the most characteristic alterations of diabetic nephropathy is the accumulation of mesangial extracellular matrix (ECM) [3]. While both short- and long-term exposure of mesangial cells (MC) to high glucose (HG) result in collagen medium accumulation [4], the metabolic alterations responsible appear dissimilar. Short-term exposure primarily stimulates synthesis of ECM components. This is in great part controlled by TGF-*β* action [5, 6] and mediated by activation of multiple diacylglycerol-sensitive protein kinase-C (PKC) isoforms [4, 7]. In contrast, long-term exposure results in a persistent collagen accumulation that is mainly due to reduced catabolism. Further, this is singularly PKC-*δ*-driven and TGF-*β*-independent [4].

Sulfonylureas close the plasma membrane ATP-sensitive K^+^ channels (K_ATP_) resulting in the depolarization and activation of voltage-dependent calcium channels, thus triggering [iCa^2+^]-dependent reactions. The K_ATP_ channel is formed by two sets of four subunits each. One is the pore-forming, inwardly rectifying K^+^ channel (Kir6.1 or Kir6.2) and the other is the regulatory sulfonylurea receptor SUR1 or SUR2 (SUR2A and its variant SUR2B). Various combinations of Kir6.x and SURx result in large heterogeneity of K_ATP_ channels providing tissue-specific channel activity [8]. Specific channels posses distinct cellular localization, electrophysiological properties, nucleotide sensitivity, and response to diverse channel inhibitors or channel openers [9, 10]. This distinct behavior includes differences in the affinity for specific sulfonylureas [11]. Sulfonylureas freely cross the cell membrane and therefore they may bind to both the cytoplasmic side of the membrane-associated SUR and to the SUR components of intracellular K_ATP_ channels [9].

Two binding components with different affinities to Glib and pharmacological properties have been described in rat glomeruli [12]. We have also demonstrated the presence of functional plasma membrane K_ATP_ channels in rat MC [13]. Two MC channels were identified as comprised by Kir6.1 in complex with either SUR2B or with a unique mesangial SUR2 splice variant (mcSUR2B) [14]. Although the physiological role of these channels remains unknown, sulfonylureas modify MC metabolism. Acute treatment (72 hours) of MC in culture with high concentrations (1.5 mM) of the sulfonylurea tolazamide resulted in augmented GLUT1 glucose transporter expression, enhanced glucose uptake, and increased synthesis and medium accumulation of collagen [15]. These changes could be demonstrated under physiological glucose concentrations and after 12- to 14-day exposure to HG.

We have also tested the effects of treatment of MC with increasing concentrations of Glib in MC in culture exposed for long periods (8 weeks) to HG [16]. As in the animal model, Glib treatment at low concentrations prevented the HG-induced accumulation of ECM. Although the mechanism for this change was undetermined, it was proposed that a Glib enhanced matrix catabolism [16]. In the present study we explored how chronic treatment with Glib may alter HG-induced accumulation of collagen IV. 

## 2. Materials and Methods

### 2.1. Cell Culture

Primary cultures of MC were obtained from outgrowths of isolated glomeruli from Munich-Wistar rats and grown and identified as described [14]. The study was approved by the Henry Ford Health System Institutional Animal Care and Use Committee (IACUC) in accord with the National Institutes of Health Guidelines for the Care and Use of Laboratory Animals. Cells were grown in RPMI medium 1640, pH 7.4, containing 5 mM glucose, 2 mM L-glutamine, 23.8 mM NaHCO_3_, 15% FBS, 5 U/mL penicillin G, and 5 U/mL streptomycin. Cells at passages 3–6 at the initiation of the experiments were used. Eight weeks before the termination of the experiments, the medium in the 25 mM high glucose (HG) experimental groups was changed to a similar one with or without Glib (Sigma Chemical Co., St. Louis, MO, USA), while the control group was continuously grown in 5 mM glucose. Forty-eight hours before the end of the experiments media were changed to one of the same composition but containing 1% FBS. Cell layers were scraped in a nondenaturing lysis buffer [4]. Samples were homogenized, centrifuged, and the supernatant used for determination of DNA content. For protein analyses, cell layers were obtained as previously described [4].

### 2.2. RNA Extraction and Quantitative RT-PCR

Total RNA was isolated from cell homogenates using the Qiagen RNeasy Mini Kit (Qiagen Inc., Valencia, CA, USA), according to the manufacturer's instructions. The measurement of gene expression in MC was by one-step RT-PCR with specific FRET probes using a multiplex mode in a LightCycler (Roche Molecular Biochemicals, Manheim, Germany). Primers and probes for *α*-2(IV) collagen and PAI-1 quantification were obtained from Roche Pharmaceuticals (Indianapolis, IN, USA) and TIB Molbiol LCC (Adelphia, NJ, USA), respectively. *β-actin* was used as reference gene. 

### 2.3. Immunoblotting

Equal amounts of protein were loaded and separated on 10% SDS-polyacrylamide gels and blotted as in our previous studies [4]. MMP-2 and *β* tubulin were identified by reaction with the following antibodies: monoclonal mouse anti-MMP-2 (Chemicon, Temecula, CA, USA), and polyclonal rabbit *β* tubulin (Santa Cruz). Immunoreactive bands were visualized with ECL detection reagent and exposure to Hyperfilm (GE Healthcare, UK). Band densities were quantified by scanning and normalizing the densitometric values to that of *β* tubulin.

### 2.4. Enzyme-Linked Immunosorbent Assay (ELISA)

The concentration of collagen IV in 48-hour conditioned media was determined in 96 well plates (Microfluor 2, Thermoelectron Corporation, Milford, MA, USA) by a high sensitivity direct ELISA [4]. Conditioned media PAI-1 protein was measured using a commercially available ELISA kit (Aniara, Mason, OH, USA) following the manufacturers' instructions.

### 2.5. Enzyme Immunoassay for Cellular cAMP

MC were seeded in 96-well plates and grown for 28 hours in 15% FBS followed by an 18-hour period in 1% FBS. Cultures were studied before and after treatment with the adenylyl cyclase activator forskolin with and without pretreatment with the nonselective phosphodiesterase (PDE) inhibitor 3-isobutyl-1-methylxanthine (IBMX, 0.5 mM). Intracellular cAMP was determined using the Direct Biotrak Enzyme Immunoassay system of Amersham Biosciences (Piscataway, NJ, USA). 

### 2.6. Protein Kinase-A (PKA) Activity Assay

PKA activity determinations were carried out using a commercially available kit (Stress X press, Assay Designs, Ann Arbor, MI, USA) according to the manufacturer's instructions. Nonspecific activity was measured by the addition of 10 *μ*M peptide inhibitor of cAMP-dependent protein kinase (PKI) that is selective for the catalytic subunit of PKA [17].

### 2.7. Zymography

MC were seeded in 6-well plates and grown for 72 hours and then were replaced by 1 mL/well of media lacking FBS and containing 0.2% lactalbumin. Conditioned media was obtained at 24 hours and 48 hours. Zymography was carried out as described [18]. Results were factored by the DNA content in the corresponding culture well.

### 2.8. Total Protein and DNA Assays

Protein measurement was carried out by a modified Lowry method that is free of interference from lysis buffer components [19]. DNA was measured by a fluorescence method using the PicoGreen dsDNA kit (Molecular Probes, Inc., Eugene, OR, USA) according to the manufacturer's directions.

### 2.9. Statistical Analysis

Statistical analyses were carried out using the StatView 5.0.1 software (Abacus Concepts Inc., Berkeley, CA, USA). Except where indicated, results were expressed as means ± SE. Significance was determined by ANOVA and differences between groups were determined by a Fisher's protected least significant difference test post-hoc. Statistical significance was set at the 5% level.

## 3. Results and Discussion

### 3.1. Effects of Glib on the HG-Enhanced Collagen IV and PAI-1 mRNA Expression and Protein Accumulation

**Figure 1:**
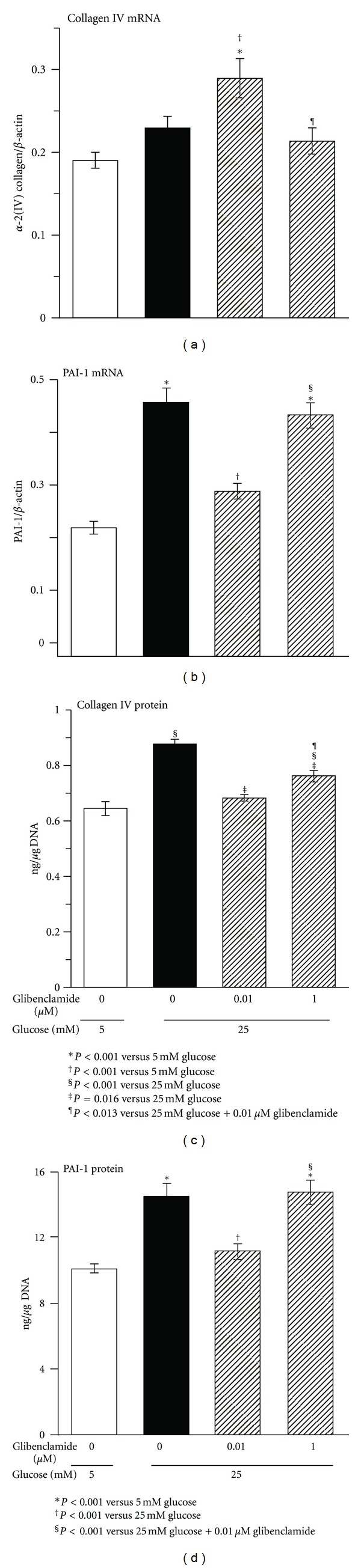
Gene and protein expression of collagen IV and PAI-1 in rat mesangial cells after chronic exposure (8 weeks) to high glucose with and without treatment with glibenclamide. The PAI-1 assay used reacts with latent, active, and inactive PAI-1. Results were factored by number of cells according to DNA content in the cell layer of the corresponding sample. mRNA *n* = 17 per experimental group, protein *n* = 12 per experimental group.

Previous work by us and others in MC has shown brisk synthesis and breakdown of newly formed collagen, achieving steady-state medium concentrations within 24 hours [20, 21]. Therefore, the results presented here in 48-hours conditioned medium reflect collagen turnover activity under steady-state conditions. In the medium of MC chronically exposed to HG, collagen IV protein accumulated 36% over values in 5 mM glucose controls (Figure 1). However, this was not associated with a significant increase in collagen IV mRNA indicating, as previously suggested for collagen I [4], that it could be the result of decreased catabolism. Chronic treatment with low-concentration Glib (0.01 *μ*M) in cells grown in HG increased collagen IV mRNA expression over that in both 5 mM controls and HG-exposed cells. Paradoxically, however, this treatment resulted in reduction in protein medium accumulation to control levels (Figure 1). This suggests enhanced turnover, that is, while Glib stimulates collagen formation it also enhances its breakdown to a greater extent. As previously shown [16], Glib demonstrated a biphasic effect at high medium concentrations (1.0 *μ*M) with unchanged collagen IV mRNA expression and attenuated decrease in protein accumulation (Figure 1). Previous dose-response studies demonstrated similar results for collagen I with intermediate effects with 0.1 *μ*M Glib [16].

We have demonstrated increased glucose transport and/or enhanced GLUT1 expression in MC treated with either high or low concentrations of sulfonylureas [15, 16]. Since GLUT1 overexpression and increased glucose transport in MC leads to exaggerated collagen I and collagen IV protein synthesis and accumulation [22], it is likely that the Glib-stimulated collagen IV mRNA is related to increased glucose uptake and associated with enhanced collagen IV protein synthesis.

PAI-1 is the primary inhibitor of plasminogen activators (PA) thus interfering with the generation of plasmin and its activation of MMP [23]. Because PAI-1 is increased in pathological conditions such as glomerulosclerosis and kidney fibrosis, the PA/PAI-1 reaction has been proposed as a novel therapeutic option for prevention and treatment of chronic kidney diseases [24]. Renal PAI-1 mRNA is overexpressed in experimental diabetes [25] and PAI-1 deficiency slows the progression of the renal disease [26, 27]. Further, HG upregulates PAI-1 protein expression in MC [28].

As in previous studies [16], HG induced MC overexpression of PAI-1 mRNA with parallel increases in protein (Figure 1). This overexpression was nullified by 0.01 *μ*M Glib (Figure 1), supporting the premise that the Glib prevention of the HG-induced collagen IV accumulation was due to increased collagen IV catabolism. However, high Glib concentration was without effect on the HG-enhanced PAI-1 mRNA and protein expression (Figure 1).

Notably, the effects of 0.01 *μ*M Glib on MC collagen IV metabolism were obtained at a concentration that is equal or less than that achieved in humans treated with low doses of the drug. In humans, following an oral dose of 1.75 mg or 7 mg (usual therapeutic range 1.25–20 mg/day), peak plasma levels at 90–120 minutes are 0.251 *μ*M and 0.686 *μ*M, respectively [29, 30]. Therefore, the results obtained in Glib-treated MC in culture are clinically relevant.

### 3.2. Effects of HG and Glib on Cellular cAMP

**Figure 2:**
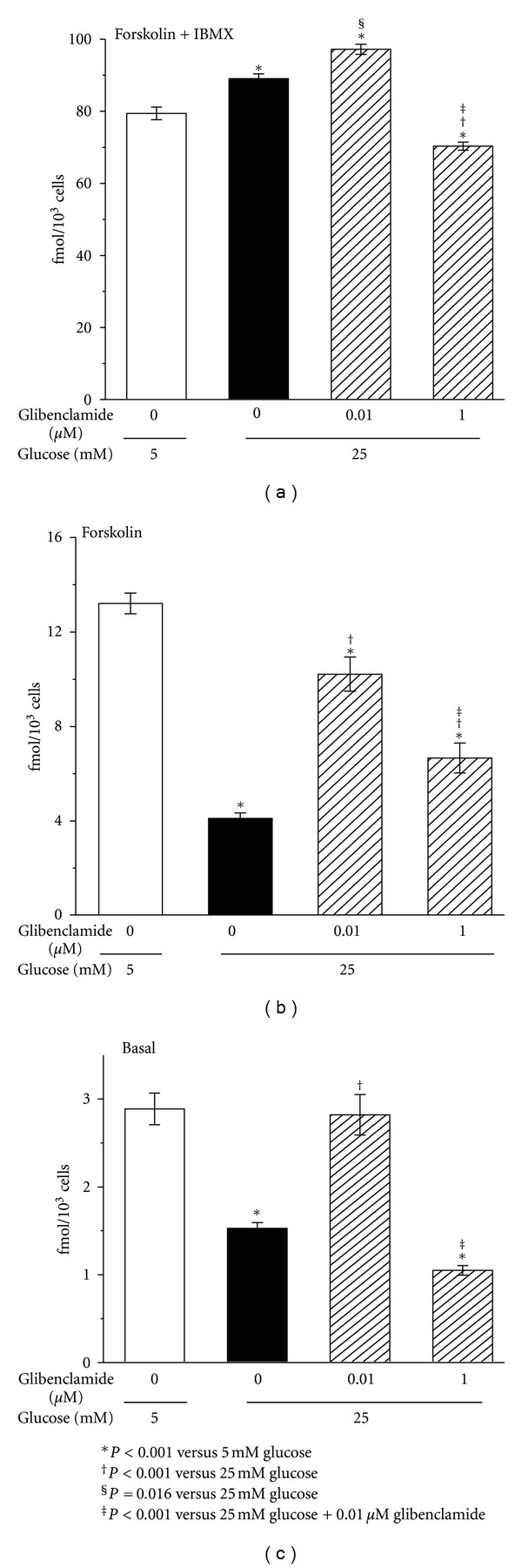
Total intracellular cAMP content in rat mesangial cells after chronic exposure (8 weeks) to high glucose with and without treatment with glibenclamide. Determinations were done under basal conditions and after 5 minutes treatment with 10 *μ*M of the adenylyl cyclase activator forskolin, with or without a 15-minute preincubation with 0.5 mM phosphodiesterase inhibitor 3-isobutyl-1-methylxanthine (IBMX) to evaluate the role of PDE activity on cAMP accumulation. *n* = 7 per experimental group.

Cyclic nucleotide analogs or agents that elevate cellular cAMP have been demonstrated to downregulate PAI-1 activity and/or PAI-1 mRNA expression in numerous cell culture systems [31, 32]. Under steady-state basal conditions MC exposed to HG contained one-half the amount of cAMP than controls, and treatment with 0.01 *μ*M Glib fully prevented this change (Figure 2). Stimulation of cAMP formation with forskolin increased cAMP 4.5-fold in controls but only 2.7-fold in HG-exposed cells. In HG conditions, 0.01 *μ*M Glib resulted in a forskolin stimulation of cAMP well above that in nontreated, HG-exposed cells (3.6-fold) but without reaching the stimulated levels in untreated controls (Figure 2). In controls, forskolin treatment during inhibition of cAMP degradation with IBMX resulted in 27-fold greater cAMP content over basal values. In HG-exposed cells, this inhibition of cAMP degradation induced a greater increase (59-fold) to values above control (Figure 2). Finally, in HG-exposed, 0.01 *μ*M Glib-treated cells, inhibition of cAMP degradation during forskolin stimulation resulted in a cAMP increase to levels above both untreated control and untreated, HG-exposed cells under the same conditions (Figure 2). In HG-exposed, 1.0 *μ*M Glib-treated cells, basal, stimulated and degradation-blocked cAMP values were in all cases lower that in 0.01 *μ*M Glib-treated cells. 

The results above suggest that chronic exposure to HG induces depressed cAMP generation that is caused by enhanced PDE activity since its inhibition during adenylyl cyclase activation restores cAMP to levels above controls. As shown by the effects of forskolin and IBMX, the presence of 0.01 *μ*M Glib prevents the HG-induced changes by restoring adenylyl cyclase synthetic activity and modulating PDE degradation of cAMP. Therefore, these findings are compatible with a Glib-induced increase in cAMP bioavailability that downregulates PAI-1.

### 3.3. Effects of HG and Glib on PKA Activity

The effects of HG and Glib on PKA activity were next investigated because although PKA is the major target of cAMP-regulated signal transduction reactions, there are also pathways that are PKA-independent [33].

**Figure 3:**
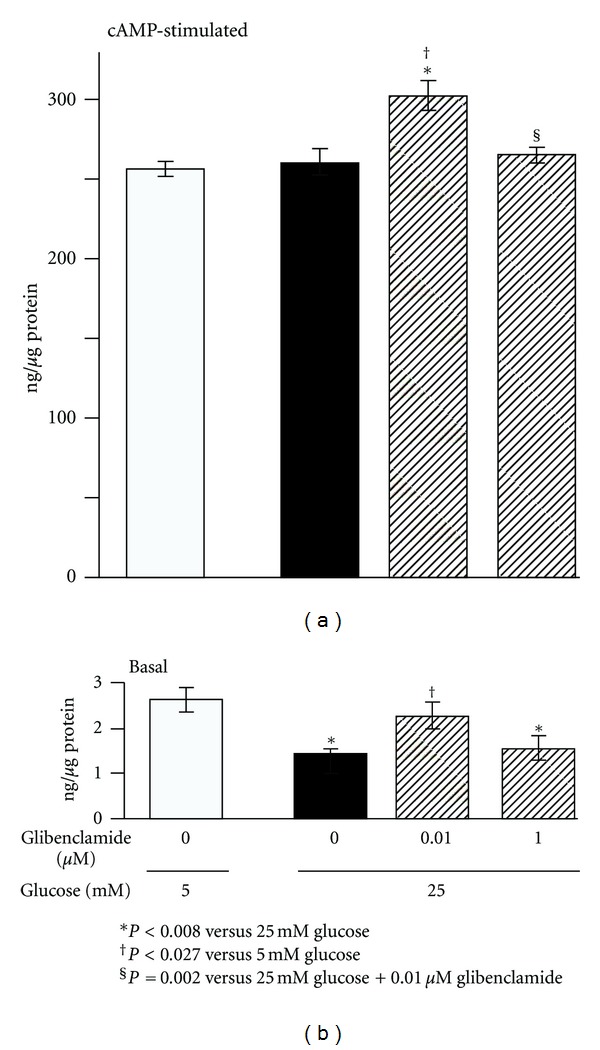
Total intracellular cAMP-dependent PKA phosphorylating activity in rat mesangial cell lysates after chronic exposure (8 weeks) to high glucose with and without treatment with glibenclamide. Determinations were done under basal conditions and after 60 minutes treatment with 20 *μ*M of cAMP. Results are the difference between total phosphorylating activity and the phosphorylating activity in duplicate samples analyzed in the presence of 10 *μ*M peptide inhibitor of cAMP-dependent protein kinase (PKI). *n* = 7 per experimental group.

cAMP-dependent PKA activity was assessed as the PKI-inhibitable phosphorylating activity under basal conditions and after stimulation with exogenous cAMP. Basal PKA activity was reduced in cells exposed to HG while treatment with 0.01 *μ*M Glib completely prevented this change (Figure 3). cAMP increased PKA activity in all groups by at least 97-fold (Figure 3). In HG exposed cells, this stimulation increased activity to levels similar to those in stimulated controls. However, the presence of 0.01 *μ*M Glib induced an additional increment that was significantly above control and HG groups (Figure 3). Treatment with 1.0 *μ*M Glib did not alter the change in PKA activity induced by HG (Figure 3). These results indicate that in HG conditions, low concentration Glib maintains a normal basal PKA activity and under conditions of activation, it permits an added cAMP-dependent PKA phosphorylating capacity.

The findings above suggest that the repression of PAI-1 expression and associated prevention of the HG-induced collagen accumulation induced by low concentrations of Glib is mediated by the activity of a cAMP-dependent PKA pathway.

### 3.4. Effects of HG and Glib on MMP2 Expression and Gelatinolytic Activity

To demonstrate that the Glib-induced decrease in PAI-1 expression subserved the decrease in collagen IV accumulation, the expression of MMP2 and its gelatinolytic capacity were determined. MMP2 (72-kDa gelatinase A) is the principal MMP formed by MC in situ and in tissue culture and mesangial ECM degradation depends on the activity of the tPA-plasmin-MMP2 cascade [34, 35].

**Figure 4:**
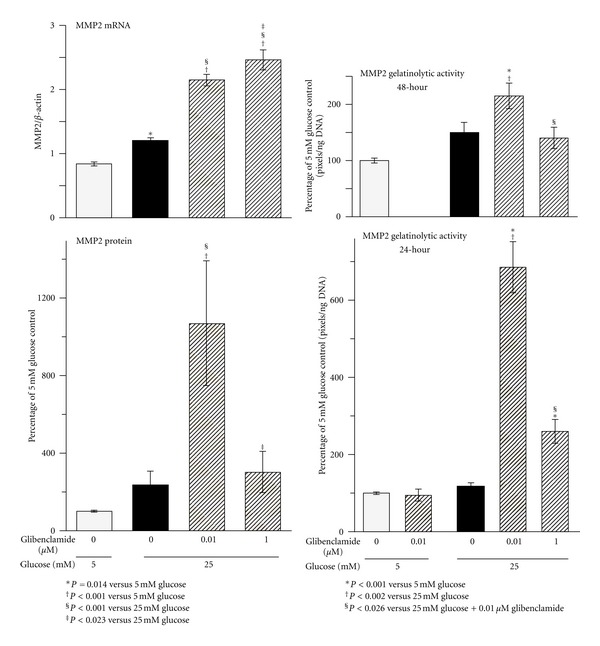
MMP2 mRNA, protein expression, and gelatinolytic activity in rat mesangial cells after chronic exposure (8 weeks) to high glucose with and without treatment with glibenclamide. Gelatinolytic activity was measured in 24- and 48-hour conditioned media. Protein and gelatinolytic activities are expressed relative to control. Zymography results were expressed as total amount of MMP2 determined by the sum of the area under both MMP2 and pro-MMP2 bands. mRNA *n* = 12, protein *n* = 4, zymography *n* = 12, per experimental group.

**Figure 5:**
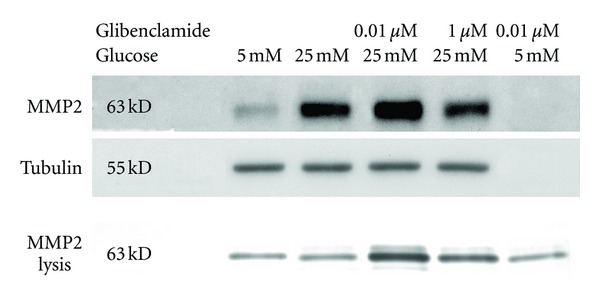
Representative immunoblotting and zymography in samples obtained from rat mesangial cells after chronic exposure (8 weeks) to high glucose with and without treatment with glibenclamide. MMP2 are samples of whole cell lysates. MMP2 lysis are samples of 24-hour conditioned media. The location of standards for molecular size identification is noted.

As previously reported [36], HG induced a small increase in MC MMP2 gene expression (Figure 4). However, this did not translate into significant changes in protein expression or gelatinolytic activity (Figures 4 and 5). The presence of 0.01 *μ*M Glib in HG cultures induced marked increases in MMP2 mRNA, MMP2 protein, and gelatinolytic activity in both 24- and 48-hour conditioned media over values in control and HG-exposed cells (Figures 4 and 5). In contrast, the presence of 1.0 *μ*M Glib in HG cultures, although inducing upregulation of MMP2 mRNA, did not change protein expression and gelatinolytic activity was only modestly increased in 48-hour conditioned medium and to a lesser extent than for 0.01 *μ*M Glib-HG cultures (Figure 4). Finally, treatment with 0.01 *μ*M Glib in 5 mM glucose grown cells did not alter gelatinolytic activity (Figures 4 and 5).

In contrast to results in this study, previous work demonstrated significantly decreased MMP2 expression in HG-exposed MC [37, 38] and depressed MMP expression and activity in glomeruli of diabetic rats [39]. However, MMP2 mRNA increases are reportedly inconsistent [40, 41]. We and others have also shown that MC exposed to HG increase ECM turnover and degradation but with a net balance of accumulation [6, 35]. In any event, multiple interventions that ameliorate diabetic nephropathy are associated with increased MMP2 activity, including treatment with angiotensin converting enzyme inhibitors [42], angiotensin II receptor antagonists [43], peroxisome proliferator-activated receptor-*γ* agonists [41], and 17*β*-estradiol [44].

Studies have shown that the cAMP analogue 8-bromo-cAMP increased MMP2 mRNA levels in cultured rat mesangial cells [45]. In addition, forskolin reportedly increased MMP2 mRNA in JAR human choriocarcinoma cells [46]. Although tPA-plasmin-MMP2 cascade plays a pivotal role in mesangial ECM degradation, we cannot exclude the possibility that low concentration Glib-induced increase in cAMP could directly activate MMP2, which needs further investigation. 

Notably, all the demonstrated metabolic effects of Glib leading to the prevention of HG-induced collagen IV accumulation were minimized or not evident using 100-fold greater concentration of the sulfonylurea. This biphasic effect of Glib is not uncommon in the mode of action of some drugs [47]. In the case of sulfonylureas, it is likely a consequence of the marked heterogeneity of K_ATP_ channels, their diverse sulfonylurea affinities, and unique metabolic actions [8]. MC contain at least two distinct K_ATP_ channels, thus, different Glib concentrations may stimulate different sets of channels and elicit divergent responses. Finally, demonstrating reversal of the effects of low concentration Glib by concomitant treatment with agents that acutely induce cAMP depletion or PKA inhibition could provide proof of cause-and-effect. However, this was not attempted because of interpretative problems arising from specificity of action and cell viability after the required 8-week period of continuous treatment.

## 4. Conclusions

Long-term treatment of MC with low concentration Glib prevents the HG-induced medium accumulation of collagen IV. This effect is likely mediated by a cAMP-PKA-dependent pathway causing inhibition of PAI-1 expression and concomitant activation of MMP2. Remarkably, low Glib concentration has little metabolic effect in MC cultured under physiological glucose conditions. It is hypothesized that the action of Glib is via tissue-specific mesangial K_ATP_ channels. Because the effect of Glib occurs with concentrations/dosages of the drug within the low range of the human therapeutic values, the findings presented here bear high clinical significance.
